# Cell-based, cell-cultured, cell-cultivated, cultured, or cultivated. What is the best name for meat, poultry, and seafood made directly from the cells of animals?

**DOI:** 10.1038/s41538-023-00234-x

**Published:** 2023-12-06

**Authors:** William K. Hallman, William K. Hallman, Eileen E. Hallman

**Affiliations:** 1 Department of Human Ecology, Rutgers, the State University of New Jersey, 55 Dudley Rd, New Brunswick, NJ 08901 USA; 2 Hallman and Associates, Rocky Hill, NJ 08553 USA; 3 Career Development & Experiential Education, Rutgers, the State University of New Jersey, 106 Somerset Street, New Brunswick, NJ 08901 USA

**Keywords:** Science, technology and society, Agriculture, Technology

## Abstract

To be sold in the United States, meat, poultry, and seafood products made from cultured cells must be labeled with a “common or usual name” to help consumers understand what they are purchasing. The terms “Cultured,” “Cultivated,” “Cell-Cultured,” “Cell-Cultivated,” “Cell-Based” and a control (without a common or usual name) were tested using an online experiment. Two regulatory criteria were assessed: that the term distinguishes the novel products from conventional products, and appropriately signals allergenicity. Three consumer acceptance criteria were assessed: that the term is seen as appropriate, does not disparage the novel or conventional products, nor elicit perceptions that the products are unsafe, unhealthy, or not nutritious. Each term was shown on packages of frozen Beef Filets, Beef Burgers, Chicken Breasts, Chicken Burgers, Atlantic Salmon Fillets, and Salmon Burgers. A representative sample of 4385 Americans (18 + ) were randomly assigned to view a single product with a single term or the control. Consumers’ ability to distinguish tested terms from conventional products differed by product category. “Cultured” and “Cultivated” failed to adequately differentiate the novel products from “Wild-Caught and Farm-Raised” salmon products. “Cultivated” failed to differentiate the novel Beef Filet product from “Grass-Fed” Beef Filets. “Cell-Cultured,” “Cell-Cultivated,” and “Cell-Based” each signaled that the products were different from conventional products across the proteins, and signaled allergenicity, meeting the two key regulatory criteria. They were not significantly different on most consumer perception measures. However, “Cell-Cultured” may have slightly better consumer acceptance across the novel beef, chicken, and salmon products, recommending its universal adoption.

## Introduction

Meat, poultry, and seafood products produced through the in vitro cultivation of animal cells that are comparable to conventional products are poised to enter the marketplace^1,2^. More than 150 companies are currently involved in developing the technology worldwide, providing inputs or producing end products, with total invested capital of $2.8 billion by 2022^3^.

The first “cultivated” chicken nugget product received regulatory approval for sale in Singapore^4,5^ and regulatory processes for these products are being developed in many other markets^6^. In the United States, the US Food and Drug Administration (FDA) and the US Department of Agriculture (USDA) Food Safety and Inspection Service (USDA-FSIS) have formally agreed to jointly regulate cell-cultured meat and poultry products. Seafood products are to be regulated solely by the FDA^7,8^.

In November 2022, the FDA completed its first pre-market consultation for a human food product made using cultured chicken cells. After evaluating the information provided to the agency by the petitioner, the FDA issued a statement that it had “no further questions at this time about the firm’s safety conclusion”^9^. A second pre-market consultation was completed in March 2023 with “no further questions,” again for a food product made using cultured chicken cells^10^. While the voluntary pre-market consultation is not an approval process and the food must meet other Federal regulatory requirements, it is a first step toward entry into the U.S. Market. In June 2023, the USDA announced that it issued grants of inspection to Upside Foods, Good Meat and Good Meat’s manufacturing partner, Joinn Biologics, bringing the products closer to being sold in restaurants and grocery stores in the U.S.^11^

Both FDA regulations (21CFR101.3) and USDA regulations for meat (9CFR317.2) and poultry products (9CFR381.117) call for the use of “common or usual names” to inform consumers about the identities of food products. As cell-cultured animal products receive regulatory approval for sale in the US and other markets, a common term will be necessary to label them and to refer to them in marketing materials.

Anticipating the need for a common or usual name for cell-based seafood products, in 2020, the US Food and Drug Administration (FDA) requested public comments on how seafood products made from the cells of fish should be labeled (85 FR 63277). Most respondents encouraged the FDA to require product identity statements that would clearly delineate cell-cultured seafood products from conventional farmed and wild-caught product, with many in the industry supporting the term “cell-cultured” seafood or “cell-based” seafood^12^, citing two studies on consumer perceptions of potential labeling terms by Hallman and Hallman^13,14^. These two terms and the five criteria used to determine them have received joint support from the main industry organization of producers of foods comprised of cultured meat, poultry, and seafood cells and the conventional seafood industry (The Alliance for Meat, Poultry and Seafood Innovation and The National Fisheries Institute)^15^, as well as from the Center for Science in the Public Interest^16^, and the Environmental Defense Fund^17^.

Consistent use of a single term to describe and to label cell-based meat, poultry, seafood, and other proteins would create greater transparency in the marketplace, help shape public perceptions, and support a greater understanding of cell-based products by consumers who would be able to use a common search term to find accurate information online. The same term would also ideally be adopted across markets to facilitate international trade and unified regulatory oversight.

There is still no unanimity regarding the choice of term^18,19^. In contrast to the apparent growing consensus regarding “cell-cultured” seafood, some in the meat industry currently support the term “cultivated” for their products, in part because it is the “best term for consumer acceptance”^20^.

While consumer acceptance is critical to the success of the industry, the common or usual name chosen to label cell-based products must meet regulatory criteria, not just marketing needs. Names chosen with the single goal of maximizing consumer acceptance^21^ may fail to meet regulatory requirements that it convey the identity of the product in a way that clearly differentiates it from other foods, and that it not be easily confused with the name of another food that is not in the same category. Failure to do so may be deemed by regulators as misleading to consumers, in violation of the Federal Food, Drug, & Cosmetic Act (FDCA) mandate that all labeling must be truthful and not misleading (21 U.S.C. § 343(a)).

Building on the Hallman and Hallman^13,14^ studies on the nomenclature for cell-based seafood products, this study uses an experimental design to evaluate potential common or usual names that would be appropriate for use in labeling cell-based meat, poultry, as well as seafood. Balancing both the need for consumer acceptance necessary for producers to sell their products and relevant regulatory requirements, Hallman and Hallman^13,14^ proposed five criteria for choosing a common or usual name.

Criterion A is that the term should enable consumers to distinguish cell-based products from conventionally produced products. Both FDA regulations (21CFR101.3) and USDA regulations for meat (9CFR317.2) and poultry products (9CFR381.117) call for the use of “common or usual names” to inform consumers about the identities of food products so that they know what they are buying and are not misled. The general principles for establishing the common or usual name of a food under 21CFR102.5 include: “The common or usual name of a food, which may be a coined term, shall accurately identify or describe, in as simple and direct terms as possible, the basic nature of the food or its characterizing properties or ingredients. The name shall be uniform among all identical or similar products and may not be confusingly similar to the name of any other food that is not reasonably encompassed within the same name. Each class or subclass of food shall be given its own common or usual name that states, in clear terms, what it is in a way that distinguishes it from different foods.” In addition, the Federal Food, Drug, & Cosmetic Act (FDCA) requires that all labeling must be truthful and not misleading (21 U.S.C. § 343(a)). Lobbying groups for conventional producers, such as the National Cattlemen’s Beef Association (NCBA) have also urged the adoption of nomenclature for “meat grown from the cells of animals” that “provides a clear and unambiguous description that effectively distinguishes the product from traditionally harvested meat”^22^.

In choosing a common or usual name for cell-based seafood products, the name should therefore make it clear to consumers that they are neither wild-caught nor farm-raised. For beef, it should distinguish cell-based products from those derived from grass-fed and conventional grain-fed cattle. For chicken, it should distinguish cell-based products from those that come from free-range chickens and those raised indoors.

Because cell-based meat, poultry, and seafood products will contain the same allergenic proteins as those in their conventional counterparts, Criterion B is that the term communicate that those allergic to these proteins should not eat the product. Food allergens remain the primary cause of anaphylaxis^23^. However, there is no cure for food allergies, and “successful avoidance depends on having complete and accurate information on food labels”^24^.

FALCPA, the Food Allergen Labeling and Consumer Protection Act of 2004 (Public Law 108-282) requires that foods containing a protein from a “major food allergen,” declare that allergen’s presence on its label. For fish and shellfish, FALCPA also requires that the specific species be named. However, allergen declarations often lack prominence on a label, and any name that makes it unclear to consumers that the cell-based products are made directly from the cells of animals to which they are allergic may negate such warnings. This is perhaps most salient with respect to seafood, to which approximately 2.3% of the US population is allergic^25^, though small numbers of Americans are allergic to meat^26^ and to poultry^27^.

In addition to meeting regulatory requirements, Hallman and Hallman^13,14^ also argued that to be readily adopted by food manufacturers, a common or usual name should help facilitate the marketing and sales of cell-based products. Criterion C is that a name should not disparage cell-based products, eliminating candidate terms such as “lab-grown,” synthetic,” “artificial,” and “fake.” Similarly, the name should not disparage the conventional products to which they might be compared, ruling out terms such as “clean,” “slaughter free,” and “cruelty-free.” Criterion D is that the term should not elicit consumer perceptions that cell-based products are unsafe, unhealthy, or less than nutritious.

Finally, a common or usual name must also satisfy the desire of consumers for transparency in food labeling^28^. Therefore, Criterion E is that consumers endorse the name as appropriate to identify the product.

Critically, a common or usual name cannot rely on pre-existing consumer knowledge and must convey meaning on its own, without any additional explanatory material on the label. Yet, most consumers have little familiarity even with the concept of producing just the parts of animals that people eat, without having to raise and harvest the entire animal^13^. Without any cell-based meat, poultry, or seafood products currently for sale in the United States, only a select few have seen or tasted them. When they do enter the marketplace, the first interaction most consumers will likely have with the common or usual name for these products will be to see it on a restaurant menu, on the label of a package at their local grocery store, or while shopping online. However, other studies of nomenclature for these products have typically provided an explanation of the technology prior to asking participants about the appeal and preferences for names to label the technology^29^.

### Nomenclature and meat, poultry, and seafood products tested and hypotheses

In this study, we test five potential common or usual names: (1) “Cell-Based,” the best term as determined from the prior Hallman and Hallman^13,14^ nomenclature studies for cell-based seafood; (2) “Cell-Cultured,” a close second best term as shown in the Hallman and Hallman^13,14^ seafood studies and preferred by the companies poised to introduce seafood products made using the cells from fish; (3) “Cultivated,” the term being championed by some supporting the introduction of meat and poultry products made from the cells of animals; (4) “Cultured,” the term used in the USDA’s Advanced Notice of Proposed Rule Making (ANPR) seeking comments on the “labeling of meat or poultry products comprised of or containing cultured animal cells” (Docket FSIS-2020-0036); and (5) “Cell-Cultivated,” a term that includes the word “cell,” which Hallman and Hallman^13^ found essential in distinguishing the novel seafood products from conventional wild-caught and farm-raised products, and a term suggested by Malerich & Bryant^30^ as appropriate nomenclature for these products. These are compared to each other and to a control without any of the terms tested.

Consistent with Hallman and Hallman^13^, we hypothesize that on their own, the terms “Cultivated” and “Cultured” will fail to distinguish products made directly from the cells of animals from their conventionally raised/harvested counterparts. As with the previously tested seafood products, “Cultivated” is expected to be confused with farmed products. In contrast, “Cell-Based,” “Cell-Cultured,” and “Cell-Cultivated” are expected to signal to consumers that the products are different from conventional products and will perform similarly in doing so.

These names are tested in association with beef, chicken, and salmon products, among the most consumed types of meat, poultry, and seafood in the United States^31^. It should be noted that in the United States, seafood packages are required to indicate whether the contents are “wild” or “wild-caught” or are “farmed” or “farm-raised,” and that neither “Cultivated” nor “Cultured” are currently acceptable substitutes for these terms (7 CFR § 60.300). In contrast, there are no “wild-caught” beef and chicken products available in American markets. All are domesticated agricultural products, raised on farms or ranches, and their methods of production are not required to be disclosed on a label. Apart from beef voluntarily labeled as “grass-fed” and chicken products voluntarily labeled as “free-range,” the methods of production of these products do not typically appear on packages of beef and chicken. However, to compensate for their additional costs of production, both “grass-fed” beef and “free-range” chicken demand price premiums above their conventionally produced counterparts, which are “grain-fed” beef (typically finished on feedlots) and chicken “raised indoors” (i.e., in chicken houses). However, only about one percent of the total beef market in the U.S. is comprised of grass-fed products that are voluntarily labeled and marketed as such, much of which is sold directly by ranchers to consumers^32^. Therefore, while most packages of beef in the U.S. almost certainly contain “grain-fed” beef, they do not bear a label indicating this. Similarly, less than one percent of chickens in the U.S. are raised as “free range”^33^, so most packages of chicken in the U.S. almost certainly contain chicken “raised indoors,” although they are not labeled accordingly. Because there is widespread “agricultural illiteracy” among Americans^34,35^, and products labeled as “grass-fed” and “free-range” make up such a small proportion of the market, methods of production for beef and chicken products may be less salient to consumers than those related to seafood. Therefore, we hypothesize that the introduction of *any* new common or usual name shown on the label of products made from the cells of beef or chicken may serve as a strong signal to consumers that the product is somehow “different” from the conventional products with which they are already familiar.

Each common or usual name is shown on packages of frozen Beef Filets, Chicken Breasts, and Atlantic Salmon Fillets. These represent familiar forms of “whole muscle cuts,” that are currently beyond the ability of the cell-based food industry to produce. The tested names are also shown on packages of frozen Beef Burgers, Chicken Burgers, and Salmon Burgers. Ground or flaked products able to be formed into burgers are less challenging to produce and are likely to be available to consumers more immediately.

In the tests of common or usual names for seafood made from the cells of fish by Hallman and Hallman^13,14^, the labels shown to consumers listed the acceptable market name of the fish congruent with the FDA’s Seafood List^36^ (e.g., Atlantic Salmon) and its form (e.g., Fillet), followed by the common or usual name tested (e.g., “Cell-Based Seafood”). Following the same pattern, a product might logically be labeled as “Beef Filets, Cell-Based Beef.” However, repeating the type of meat (e.g., “beef”) as part of a common or usual name is inconsistent with current USDA regulations and using the generic term “Cell-Based Meat” may suggest that the product is made from a protein other than beef. Moreover, use of the term “meat” may not be consistent with cultural meanings, regulatory requirements, or with halal or kosher labeling in some regions of the world^6^. Therefore, we test labels following the format of “Beef Filets, Cell-Based.”

## Results

### Description of participants and random assignment to conditions

The median length of the experiment was 12.4 min. Consistent with census data, 53.1% of the 4385 participants were female. Mean age was 44.91, SD = 17.86; 35.6% reported children under the age of 18 and 21.6% reported children under age 5 lived in their households. When asked “who does the grocery shopping for the household,” 54.3% reported doing “all of it,” 18.2% “most of it,” 17.4% “about half of it,” 6.9% “some of it,” 2.5% “someone else does all of it,” and 0.7% preferred not to answer. Additional sociodemographic characteristics of the participants are shown in Table 1. After random assignment, 733 participants saw the Control (just the product without any term tested). The remaining participants saw products labeled as “Cell-Based” (*n* = 711), “Cell-Cultured” (*n* = 734), “Cell-Cultivated” (*n* = 729), “Cultured” (*n* = 729), or “Cultivated” (*n* = 749). Beef Filets were seen by 719 participants, Beef burgers (*n* = 733), Chicken Breasts (*n* = 738), Chicken Burgers (*n* = 731), Salmon Fillets (*n* = 733), Salmon Burgers (*n* = 731).

**Table 1:** Sociodemographic Characteristics of the Sample, (*N*) = 4385.

Sociodemographic characteristics	% of total
Marital status	
Married	49.2%
Single, never married	29.0%
Divorced or separated	9.4%
Living with partner	8.9%
Widowed	3.8%
Prefer Not to Disclose	0.7%
Educational level	
Less than high school	3.2%
High school /GED	23.5%
Some college	23.4%
2-year college degree (Associate)	11.0%
4-year college degree (BA, BS)	22.9%
Graduate or Professional Degree	14.6%
Doctoral Degree	1.4%
Census Region	
Northeast	18.0%
Midwest	20.0%
South	42.4%
West	19.6%
Race/Ethnicity	
Non-Hispanic White	62.2%
Non-Hispanic Black/African American	15.4%
Hispanic/Latino	10.6%
Non-Hispanic Asian	6.6%
Non-Hispanic Two or More Races	2.6%
Non-Hispanic Other	2.6%
Household income	
Below $25,000	17.3%
$25,000–$49,999	22.6%
$50,000–$74,999	18.0%
$75,000–$99,999	15.9%
$100,000–$149,999	15.9%
$150,000–$199,999	6.1%
$200,000 or more	4.2%

Of the 1452 participants randomly assigned to view a beef product, 96.2% reported having eaten one or more meals containing beef in the 12 months prior to the survey. Of the 1469 participants who viewed a chicken product, 97.5% had eaten at least one meal containing chicken in the prior 12 months. Of the 1464 participants assigned to see a salmon product, 77.0% indicated that they had eaten at least one meal containing salmon in the prior year.

Asked to rate their familiarity with the product they saw, the participants were moderately to very familiar with Chicken Breasts (*M* = 3.88, SD = 1.15), beef burgers (*M* = 3.53, SD = 1.31), Salmon Fillets (*M* = 3.20, SD = 1.30), and Beef Filets (*M* = 3.02, SD = 1.30). They were only slightly to moderately familiar with Chicken Burgers (*M* = 2.68, SD = 1.32) and salmon burgers (*M* = 2.33, SD = 1.34) [Scale: 1 not at all; 2 slightly; 3 moderately; 4 very; 5 extremely].

Initial tests of the five criteria reported in sections 3.1–3.4 below were conducted without explaining to the participants the meaning of the terms they saw on the packages.

### Criterion A—Ability to distinguish from conventional products

An essential function of a new common or usual name is to signal that the product bearing it is different from the products with which consumers may already be familiar. Z-tests for equality of proportions using a Bonferroni correction were used to examine differences in the ability of each of the common or usual name to communicate to the participants that the product was different from its conventional counterpart (Tables 2a–4b).

**Table 2:** **a** Percent Describing the Beef Filets as Grass-Fed, Grain Fed, or Neither, By Common or Usual Name. **b** Percent Describing the Beef Burgers as Grass-Fed, Grain Fed, or Neither, By Common or Usual Name Tested and Control.

	Cell-Based	Cell-Cultured	Cell-Cultivated	Cultured	Cultivated	|Control|
**a**						
*Beef Filets*						
Neither Grass-Fed nor Grain-Fed	76.7%_a_	70.3%_a,b_	78.0%_a_	68.6%_a,b_	57.0%_b_	76.6%_a_
Grass-Fed	18.3%_a,d,e_	22.9%_a,d,e_	14.4%_a,b_	14.4%_a,c_	33.9%_d_	13.7%_b,c,e_
Grain-Fed	5.0%_a_	6.8%_a,b_	7.6%_a,b_	16.9%_b_	9.1%_a,b_	9.7%_a,b_
*N*	120	118	118	118	121	124
**b**						
*Beef Burgers*						
Neither Grass-Fed nor Grain-Fed	76.5%_a_	73.2%_a_	75.6%_a_	70.2%_a_	68.5%_a_	75.8%_a_
Grass-Fed	15.1%_a_	14.2%_a_	16.0%_a_	20.2%_a_	23.4%_a_	15.8%_a_
Grain-Fed	8.4%_a_	12.6%_a_	8.4%_a_	9.7%_a_	8.1%_a_	8.3%_a_
*N*	119	127	119	124	124	120

Values in the same row not sharing the same subscript are significantly different at *p* < 0.05 in the two-sided test of equality for column proportions using the Bonferroni correction. Total *N* = 719.

Values in the same row not sharing the same subscript are significantly different at *p* < 0.05 in the two-sided test of equality for column proportions using the Bonferroni correction. Total *N* = 733.

**Table 3:** **a** Percent Describing the Chicken Breasts as Free-Range, Raised Indoors, or Neither, By Common or Usual Name Tested and Control. **b** Percent Describing the Chicken Burgers as Free-Range, Raised Indoors, or Neither, By Common or Usual Name Tested and Control.

	Cell-Based	Cell-Cultured	Cell-Cultivated	Cultured	Cultivated	|Control|
**a**						
*Chicken Breasts*						
Neither Free-Range nor Raised Indoors	67.2%_a_	67.5%_a_	59.5%_a_	65.8%_a_	60.3%_a_	72.5%_a_
Free-Range	18.8%_a_	19.5%_a_	30.2%_a_	19.2%_a_	16.0%_a_	19.2%_a_
Raised Indoors	14.1%_a,b_	13.0%_a,b_	10.3%_a,b_	15.0%_a,b_	23.7%_a_	8.3%_b_
*N*	128	123	116	120	131	120
**b**						
*Chicken Burgers*						
Neither Free-Range nor Raised Indoors	65.2%_a,b_	63.9%_a,b_	66.1%_a,b_	58.6%_a_	58.5%_a_	76.2%_b_
Free-Range	16.5%_a_	23.5%_a_	17.7%_a_	28.9%_a_	14.6%_a_	15.6%_a_
Raised Indoors	18.3%_a,b_	12.6%_a,b_	16.1%_a,b_	12.5%_a,b_	26.8%_a_	8.2%_b_
*N*	115	119	124	128	123	122

Values in the same row not sharing the same subscript are significantly different at *p* < 0.05 in the two-sided test of equality for column proportions using the Bonferroni correction. Total *N* = 738.

Values in the same row not sharing the same subscript are significantly different at *p* < 0.05 in the two-sided test of equality for column proportions using the Bonferroni correction. Total *N* = 731.

**Table 4:** **a** Percent Describing the Salmon Fillets as Wild-Caught, Farm-Raised, or Neither, By Common or Usual Name Tested and Control. **b** Percent Describing the Salmon Burgers as Wild-Caught, Farm-Raised, or Neither, By Common or Usual Name Tested and Control.

	Cell-Based	Cell-Cultured	Cell-Cultivated	Cultured	Cultivated	|Control|
**a**						
*Salmon Fillets*						
Neither Wild-Caught nor Farm-Raised	70.8%_a_	56.6%_a,b_	58.0%_a,b_	49.2%_b,c_	33.1%_c_	41.3%_b,c,d_
Wild-Caught	17.7%_a_	16.3%_a_	20.2%_a_	20.2%_a_	25.2%_a_	43.8%_b_
Farm-Raised	11.5%_a_	27.1%_b,c,d_	21.8%_a,b_	30.6%_b,c_	41.7%_c_	14.9%_a,d_
*N*	113	129	119	124	127	121
**b**						
*Salmon Burgers*						
Neither Wild-Caught nor Farm-Raised	66.4%_a_	66.1%_a_	60.9%_a,b_	46.1%_b,c_	32.5%_c_	44.4%_b,c,d_
Wild-Caught	20.7%_a_	18.6%_a_	19.5%_a_	26.1%_a,b_	19.5%_a_	42.9%_b_
Farm-Raised	12.9%_a,c_	15.3%_a,c_	19.5%_a,c_	27.8%_a_	48.0%_b_	12.7%_c_
*N*	116	118	133	115	123	126

Values in the same row not sharing the same subscript are significantly different at *p* < 0.05 in the two-sided test of equality for column proportions using the Bonferroni correction. Total *N* = 733.

Values in the same row not sharing the same subscript are significantly different at *p* < 0.05 in the two-sided test of equality for column proportions using the Bonferroni correction. Total *N* = 731.

Overall, there were different patterns of results depending on the protein (beef, chicken, salmon), and the form of the product (whole cut or burger) shown to the participants. As shown in Table 2a, for Beef Filets, the common name “Cultivated” performed most poorly in signaling that the product was “Neither Grass-Fed nor Grain-Fed Beef.” Only 57.0% of the participants correctly identified the product as being different from conventional beef products and one-third (33.9%) mistakenly believed that the product was “Grass-Fed Beef.” In contrast, 76.6% of those who saw the Control product (which only 9.7% correctly interpreted as conventional “Grain-Fed Beef”) reported that the Beef Filets were “Neither Grass-Fed nor Grain-Fed Beef” and only 13.7% thought that the product was “Grass-Fed Beef.” The other terms tested performed similar to the Control in signaling that the product was “Neither Grass-Fed nor Grain-Fed Beef.”

For Beef Burgers, the pattern of results was different. There were no statistically significant differences among any of the common or usual names tested or the Control with respect to the proportions of participants who thought that the products were “Neither Grass-Fed nor Grain Fed” (Table 2b).

There were also no differences among the names tested in signaling that the Chicken Breasts were “Neither Free-Range nor Raised Indoors” and none outperformed the Control (72.5%) (which should have been interpreted as “Raised Indoors’) (Table 3a). Yet, when testing the terms on packages of Chicken Burgers, neither “Cultured” (58.6%) nor “Cultivated” (58.5%) performed better than the Control (76.2%) in signaling that the Chicken Burgers were “Neither Free-Range nor Raised Indoors” (Table 3b).

As expected, for Salmon Fillets (Table 4a), the terms “Cultivated” and “Cultured” performed least well. Only one-third (33.1%) of those who saw the term “Cultivated” thought it was “Neither Wild-Caught nor Farm-Raised” and 41.7% thought it was “Farm-Raised.” Fewer than half (49.2%) of the participants who saw the term “Cultured” responded that the product was “Neither Wild-Caught nor Farm-Raised,” while 30.6% thought they were “Farm-Raised.” In contrast, 70.8% of those who saw the term “Cell-based” correctly identified the products as “Neither Wild-Caught nor Farm-Raised,” as did 56.6% of those who saw “Cell-cultured” and 58.0% of those who saw the term “Cell-Cultivated.”

The terms “Cultivated” and “Cultured” also performed poorly for Salmon Burgers (Table 4b). “Cultivated” indicated to less than one-third of the participants (32.5%) that the salmon was “Neither Wild-Caught nor Farm-Raised” and nearly half (48.0%) thought the salmon was “Farm-Raised.” “Cultured” signaled to only 46.1% of the participants that the salmon was “Neither Wild-Caught nor Farm-Raised” and 27.8% thought it was “Farm-Raised.” In contrast, 66.4% of those who saw the term “Cell-Based,” 66.1% of those who saw “Cell-Cultured,” and 60.9% of those who saw “Cell-Cultivated” correctly identified the products as “Neither Wild-Caught nor Farm-Raised.”

A two-way ANOVA examining the effects of the names tested and the products tested on the confidence the participants had in their answers regarding whether the product was a conventional product found no significant interaction effect (*F*(5, 4349) = 1.238, *p* = 0.192). However, there was a main effect of name tested (*F*(5, 4349) = 4.816, *p* < 0.001, η_p_^2^ = 0.006). The participants who saw the Control (*M* = 2.97, SD = 1.47) and the products labeled as “Cultured” (*M* = 2.98, SD = 1.37) and “Cultivated” (*M* = 3.01, SD = 1.32) were least confident in their answers [Scale: 1 not at all; 2 slightly; 3 moderately; 4 very; 5 extremely confident]. Participants were significantly more confident in their answers when the products they saw were labeled as “Cell-Cultivated” (*M* = 3.22, SD = 1.40) and “Cell-Cultured” (*M* = 3.21, SD = 1.38) than when they were labeled as “Cultured” or “Cultivated.” The confidence of those who saw products labeled as “Cell-Based” (*M* = 3.07, SD = 1.36) was not significantly different from the confidence of those who saw any of the other terms.

There was also a main effect of the product tested (*F*(5, 4349) = 4.445, *p* < 0.001, η_p_^2^ = 0.005). Those who saw the Chicken Burgers (*M* = 2.91, SD = 1.40) were less confident in their answers than those who saw the Salmon Burgers (*M* = 3.23, SD = 1.33) or the Salmon Fillets (*M* = 3.14, SD = 1.34). Confidence was not significantly different among those who viewed the Beef Burgers (*M* = 3.09, SD = 1.38), Beef Filets (*M* = 3.07, SD = 1.44) or the Chicken Breasts (*M* = 3.02, SD = 1.42), Salmon Burgers, or Salmon Fillets.

One-way ANOVAs indicated no main effect of name tested on how likely the participant indicated they would be to search for more information about the product on the Internet (*F*(5, 4349) = 1.42, *p* = 0.213) or to scan a QR code for more information about the product (*F*(5, 4349) = 0.997, *p* = 0.430).

### Criterion B—Signal the presence of potential allergens

The ability to signal potential allergenicity is also a critical regulatory criterion. The participants were asked, “If you are allergic to Beef/Chicken/Salmon, how safe is it for you to eat these Beef Filets/Beef Burgers/Chicken Breasts/Chicken Burgers/Atlantic Salmon Fillets/Salmon Burgers?” [Scale: 1 very unsafe; 2 moderately unsafe; 3 somewhat unsafe; 4 neither safe nor unsafe; 5 somewhat safe; 6 moderately safe; 7 very safe]. A two-way ANOVA showed no interaction effects between name tested and protein tested (beef/chicken/salmon) (*F*(10, 4367) = 0.215, *p* = 0.995). There was a main effect of the protein tested (*F*(2, 4367) = 11.71, *p* < 0.001, η_p_^2^ = 0.005). The salmon products were judged less safe to consume by those allergic to salmon (*M* = 2.82, SD = 2.11) than consumption of the beef products by those allergic to beef (*M* = 3.13, SD = 2.15) or consumption of the chicken products by those allergic to chicken (*M* = 3.16, SD = 2.14).

There was also a main effect of the name tested (*F*(5, 4367) = 7.548, *p* < 0.001, η_p_^2^ = 0.009). All the names and the Control appropriately signaled that it was moderately to somewhat unsafe to eat the products if one were allergic to the protein from which they were made (Table 5). However, the Control (*M* = 2.73, SD = 2.13) and the products with the terms “Cultivated” (*M* = 2.87, SD = 2.14) were seen as least safe to consume by those allergic to the protein.

**Table 5:** How Safe to Eat If Allergic to Beef/Chicken/Salmon By Common or Usual Name Tested and Control.

	*M*	SD	*N*	*F*	*P* value	η^2^
Safe to Eat				5.63	<0.001	0.009
Cell-Based	3.26_a_	2.16	711			
Cell-Cultivated	3.23_ab_	2.07	729			
Cell-Cultured	3.17_abc_	2.11	734			
Cultured	2.96_abc_	2.18	729			
Cultivated	2.87_cd_	2.14	749			
Control	2.73_d_	2.13	733			

Scale: 1 very unsafe; 2 moderately unsafe; 3 somewhat unsafe; 4 neither safe nor unsafe; 5 somewhat safe; 6 moderately safe; 7 very safe. Means with the same superscript letter are not significantly different from each other at *p* < 0.05 using the Tukey HSD post hoc test.

### Criteria C and D—Not disparage or create false perceptions of products

The participants were asked to carefully examine the package shown to them and asked to type their response to the question, “What is the first thought, image, or feeling that comes to mind when seeing this package?” They were then asked to look at the package a second time and to record the second thought, image, or feeling that came to mind. Each of the responses was coded using one of the categories developed by Hallman and Hallman (2020) (see Tables S1 and S2 in the supplemental materials). Two trained researchers independently coded each response, with any discrepancies resolved by consensus. After recording their open-ended responses, each participant rated how positive or negative their thought, image, or feeling was, using a scale ranging from 1 extremely negative to 7 extremely positive. The participants were asked to look at the package a third time and to record how positive or negative their overall reactions were. A MANOVA examining the effects of name tested and product tested on all three ratings as dependent measures found main effects of name, *F*(15, 11998) = 2.662, *p* < 0.001; Wilk’s Λ = 0.991, η_p_^2^ = 0.003, and product, *F*(15, 11998) = 4.016, *p* < 0.001; Wilk’s Λ = 0.986, η_p_^2^ = 0.005, but no interaction *F*(75, 12993) = 1.150, *p* = 0.117; Wilk’s Λ = 0.980, η_p_^2^ = 0.007.

As shown in Table 6, the first and second thoughts, images, and feelings and overall reactions associated with the control products, and those products labeled with the terms “Cultivated” and “Cultured,” were as positive as those labeled with the terms “Cell-Based,” and “Cell-Cultured.” “Cell-Cultivated” was viewed least positively. With respect to the products, the Beef Filets garnered the most positive responses, while the Chicken Burgers received the least positive reactions (Table 7).

**Table 6:** Ratings of thoughts, images, or feelings and overall reactions by common or usual name tested and control.

	*M*	SD	*N*	*F*	*P* value	η^2^
Rating of First Thought, Image or Feeling				4.278	<0.001	0.005
Control	5.27_a_	1.65	733			
Cultivated	5.26_a_	1.60	749			
Cultured	5.23_a_	1.67	729			
Cell-Cultured	5.10_ab_	1.74	734			
Cell-Based	5.02_ab_	1.71	711			
Cell-Cultivated	4.99_b_	1.79	729			
Rating of Second Thought, Image or Feeling				4.781	<0.001	0.005
Control	5.14_a_	1.76	733			
Cultured	5.08_a_	1.74	729			
Cultivated	5.06_a_	1.68	749			
Cell-Cultured	4.88_ab_	1.79	734			
Cell-Based	4.87_ab_	1.80	711			
Cell-Cultivated	4.70_b_	1.86	729			
Overall Reactions				6.990	<0.001	0.008
Control	5.28_a_	1.70	733			
Cultured	5.21_a_	1.70	729			
Cultivated	5.19_ab_	1.68	749			
Cell-Based	4.98_bc_	1.76	711			
Cell-Cultured	4.97_bc_	1.79	734			
Cell-Cultivated	4.85_c_	1.84	729			

Scale: 1 extremely negative; 2 moderately negative; 3 slightly negative; 4 neither positive nor negative; 5 slightly positive; 6 moderately positive; 7 extremely positive.

Values in the same subtable not sharing the same subscript are significantly different at *p* < 0.05 as determined by Tukey’s HSD.

**Table 7:** Overall ratings by product.

	*M*	SD	*N*	*F*	*P* value	η^2^
Overall Rating of Product				9.807	<0.001	0.011
Beef Filets	5.30_a_	1.72	719			
Salmon Fillets	5.23_ab_	1.77	733			
Chicken Breasts	5.16_ab_	1.62	738			
Beef Burgers	5.11_ab_	1.72	733			
Salmon Burgers	4.95_b_	1.82	731			
Chicken Burgers	4.75_c_	1.80	731			

Scale: 1 extremely negative; 2 moderately negative; 3 slightly negative; 4 neither positive nor negative; 5 slightly positive; 6 moderately positive; 7 extremely positive.

Values in the same subtable not sharing the same subscript are significantly different at *p* < 0.05 as determined by Tukey’s HSD.

A MANOVA was used to explore the effects of name and product tested on the dependent measures described below. The analysis showed main effects of name, *F*(55, 2083) = 6.640, *p* < 0.001; Wilk’s Λ = 0.920, η_p_^2^ = 0.017, and product, *F*(55, 2083 = 12.696, *p* < 0.001; Wilk’s Λ = 0.854, η_p_^2^ = 0.031, but no interaction effect, *F*(275, 47828) = 1.108, *p* = 0.105; Wilk’s Λ = 0.932, η_p_^2^ = 0.006. We therefore focus on the main effects of name tested.

### Interest in tasting, likelihood of purchasing, ordering, serving

There was a significant effect of name on interest in tasting the products (*F*(5,4348) = 4.426, *p* < 0.001, η_p_^2^ = 0.005). The participants were moderately interested in tasting all the products. They were equally interested in tasting the Control and the products labeled as “Cultured,” “Cultivated” and “Cell-Cultured,” and less interested in tasting the products labeled as “Cell-Based” and “Cell-Cultivated” (Table 8).

**Table 8:** Interest in tasting by common or usual name tested and control.

	*M*	SD	*N*	*F*	*P* value	η^2^
Interest in Tasting				4.35	<0.001	0.005
Cultured	3.48_a_	1.41	729			
Control	3.47_a_	1.42	733			
Cultivated	3.42_ab_	1.41	749			
Cell-Cultured	3.31_ab_	1.44	734			
Cell-Based	3.24_b_	1.41	711			
Cell-Cultivated	3.23_b_	1.50	729			

Scale: 1 not at all interested, 2 slightly interested, 3 moderately interested, 4 very interested, 5 extremely interested. Means with the same superscript letter are not significantly different from each other at *p* < 0.05 using Tukey HSD post hoc Test.

There was a significant effect of name on reported likelihood to purchase the products in the next 6 months if it were sold in their grocery store (*F*(5,4348) = 4.346 *p* < 0.001, η_p_^2^ = 0.005). Participants were “neither likely nor unlikely” to “slightly likely” to purchase all the products. They were most likely to purchase the Control products, those labeled as “Cultured,” “Cultivated,” and “Cell-Cultured,” and slightly less likely to purchase those labeled as “Cell-Based,” or “Cell-Cultivated.” (Table 9).

**Table 9:** Likelihood to purchase by common or usual name tested and control.

	*M*	SD	*N*	*F*	*P* value	η^2^
Likelihood to Purchase				4.01	<0.001	0.005
Control	4.92_a_	2.11	733			
Cultured	4.88_ab_	2.06	729			
Cultivated	4.79_abc_	2.08	749			
Cell-Cultured	4.73_abc_	2.15	734			
Cell-Based	4.57_bc_	2.13	711			
Cell-Cultivated	4.54_c_	2.12	729			

Scale: 1 extremely unlikely, 2 moderately unlikely, 3 slightly unlikely, 4 neither likely nor unlikely, 5 slightly likely, 6 moderately likely, 7 extremely likely. Means with the same superscript letter are not significantly different from each other at *p* < 0.05 using the Tukey HSD post hoc Test.

There was a significant effect of name tested on likelihood to order the products in a restaurant (*F*(5,4348) = 4.212, *p* < 0.001, η_p_^2^ = 0.005). Participants were “neither likely nor unlikely” to “slightly likely” to order the products. They were most likely to order the “Cultured” and Control products and those labeled as “Cultivated” and “Cell-Cultured,” and slightly less likely to order those labeled as “Cell-Based” and “Cell-Cultivated” (Table 10).

**Table 10:** Likelihood to order in a restaurant by common or usual name tested and control.

	*M*	SD	*N*	*F*	*P* value	η^2^
Likelihood to Order in a Restaurant				4.21	<0.001	0.005
Cultured	4.80_a_	2.05	729			
Control	4.80_a_	2.08	733			
Cultivated	4.68_ab_	2.07	749			
Cell-Cultured	4.58_ab_	2.12	734			
Cell-Based	4.48_b_	2.16	711			
Cell-Cultivated	4.44_b_	2.20	729			

Scale: 1 extremely unlikely, 2 moderately unlikely, 3 slightly unlikely, 4 neither likely nor unlikely, 5 slightly likely, 6 moderately likely, 7 extremely likely. Means with the same superscript letter are not significantly different from each other at *p* < 0.05 using the Tukey HSD post hoc Test.

There was a significant effect of name on likelihood to serve the products to guests in the next 6 months (*F*(5,4348) = 5.052, *p* < 0.001, η_p_^2^ = 0.006). Participants were “neither likely nor unlikely” to “slightly likely” to serve the products to guests. They were most likely to serve the Control products and least likely to serve those labeled as “Cell-Based” (Table 11).

**Table 11:** Likelihood to serve to guests by common or usual name tested and control.

	*M*	SD	*N*	*F*	*P* value	η^2^
Likelihood to Serve to Guests				5.05	<0.001	0.006
Control	4.72_a_	2.13	733			
Cultured	4.64_ab_	2.12	729			
Cultivated	4.52_abc_	2.14	749			
Cell-Cultured	4.42_abc_	2.21	734			
Cell-Cultivated	4.36_bc_	2.17	729			
Cell-Based	4.25_c_	2.17	711			

Scale: 1 extremely unlikely, 2 moderately unlikely, 3 slightly unlikely, 4 neither likely nor unlikely, 5 slightly likely, 6 moderately likely, 7 extremely likely. Means with the same superscript letter are not significantly different from each other at *p* < 0.05 using the Tukey HSD post hoc Test.

### Safe to eat

All the names appropriately signaled that if one is not allergic to the protein from which they are made, it is somewhat to moderately safe to eat the products - “Cultivated” (*M* = 5.98, SD = 1.40), “Cultured” (*M* = 5.95, SD = 1.52), Control (*M* = 5.94, SD = 1.49), “Cell-Cultivated” (*M* = 5.83, SD = 1.45), “Cell-Based” (*M* = 5.79, SD = 1.54), “Cell-Cultured” (*M* = 5.75, SD = 1.55) [Scale: 1 very unsafe to 7 very safe]. However, the products with the term “Cultivated” were seen as safer than those labeled as “Cell-Cultured” (*F*(5,4348) = 3.042, *p* = 0.010, η_p_^2^ = 0.003).

### Natural, organic, genetically modified

There was a main effect of name tested on participant perceptions of the product’s naturalness [Scale: 1 very unnatural to 7 very natural] (*F*(5,4348)) = 20.887 *p* < 0.001, η_p_^2^ = 0.023). The Control (*M* = 5.25, SD = 1.51) was perceived as the most natural product. The products labeled as “Cultivated” (*M* = 5.00, SD = 1.63), and “Cultured” (*M* = 4.93, SD = 1.65) were seen as equally natural, and both were viewed as more natural than products with the terms “Cell-Cultivated” (*M* = 4.61, SD = 1.84), “Cell-Based” (*M* = 4.60, SD = 1.81) and “Cell-Cultured” (*M* = 4.51, SD = 1.85), which were seen as equally natural.

There was no main effect of name tested on participant perceptions of the likelihood that the product is organic [Scale 1 extremely unlikely to 7 extremely likely] (*M* = 4.31, SD = 1.83), (*F*(5,4348)) = 0.735 *p* = 0.597). There was a main effect of name tested on participant perceptions of the likelihood that the product is genetically modified [Scale 1 extremely unlikely to 7 extremely likely] (*F*(5,4348)) = 34.827 *p* < 0.001, η_p_^2^ = 0.039). Products with the terms “Cell-Cultivated” (*M* = 5.30, SD = 1.64), “Cell-Based” (*M* = 5.24, SD = 1.60) and “Cell-Cultured” (*M* = 5.23, SD = 1.58) were seen as more likely to be genetically modified than products with the terms “Cultured” (*M* = 4.81, SD = 1.60) and “Cultivated” (*M* = 4.79, SD = 1.63). The Control (*M* = 4.41, SD = 1.66) was seen as the least likely to be genetically modified.

### Nutritious, Healthy

After being shown the enlarged nutrition facts label, the participants were asked how nutritious they thought the products are [Scale: 1 not at all; 2 slightly; 3 moderately; 4 very; 5 extremely] and how healthy they are [Scale: 1 extremely unhealthy – 7 extremely healthy]. There was no main effect of name tested on perceptions of nutritiousness (*M* = 3.31, SD = 1.14) (*F*(5,4348)) = 0.318 *p* = 0.902) or on perceptions of healthiness (*M* = 4.98, SD = 1.60) (*F*(5,4348)) = 0.436 *p* = 0.824). Overall, the products were seen as moderately nutritious and neither healthy nor unhealthy. Perceptions of nutritiousness and healthiness were strongly correlated r(4383) = 0.74, *p* < 0.001.

### Taste

There was a significant main effect of name tested on how the participants think the product tastes [Scale: 1 extremely bad – 7 extremely good] (*F*(5,4348)) = 3.254 *p* = 0.006, η_p_^2^ = 0.004). While each product was thought to taste slightly to moderately good, those labeled as “Cultured” were thought to taste slightly better (*M* = 5.33, SD = 1.58) than those labeled as “Cell-Based” (*M* = 5.07, SD = 1.62). Post hoc tests detected no other differences; Control (*M* = 5.31, SD = 1.65), “Cultivated” (*M* = 5.29, SD = 1.56), “Cell-Cultured” (*M* = 5.20, SD = 1.61), “Cell-Cultivated” (*M* = 5.12, SD = 1.60).

### Likelihood to recommend to pregnant women and to children

A MANOVA found main effects of product tested (*F*(10, 8696) = 9.985, *p* < 0.001; Wilk’s Λ = 0.977, η_p_^2^ = 0.004) and name tested (*F*(10, 8696) = 3.084, *p* < 0.001; Wilk’s Λ = 0.993, η_p_^2^ = 0.011), on the likelihood that the participant would recommend that pregnant women eat the product and that children eat the product. There was no interaction (*F*(50, 8696) = 1.28, *p* = 0.026; Wilk’s Λ = 0.984, η_p_^2^ = 0.008).

Examining the main effect of name on likelihood to recommend that those who are pregnant eat the products, (*F*(5,4348)) = 4.043 *p* = 0.001, η_p_^2^ = 0.005), the participants reported that they would be “neither likely nor unlikely” to recommend the products be eaten by pregnant women (Table 11). They were most likely to recommend that those who are pregnant consume the Control products, and less likely to recommend the “Cell-Cultured,” “Cell-Based” and “Cell-Cultivated” products.

Similarly, the participants reported that they would be “neither likely nor unlikely” to recommend the products be eaten by children (Table 12) (*F*(5,4348)) = 4.578 *p* < 0.001, η_p_^2^ = 0.005). They were most likely to recommend that children consume the Control products and less likely to recommend the “Cell-Cultured” and “Cell-Based” products.

**Table 12:** Likelihood to recommend that pregnant women and children eat the product by common or usual name tested and control.

	*M*	SD	*N*	*F*	*P* value	η^2^
Likelihood to Recommend Pregnant Women Eat the Product				4.04	<0.001	0.005
Control	4.40_a_	1.94	733			
Cultured	4.28_ab_	1.94	729			
Cultivated	4.22_ab_	1.93	749			
Cell-Cultured	4.19_b_	1.93	734			
Cell-Based	4.04_b_	1.98	711			
Cell-Cultivated	4.02_b_	1.96	729			
Likelihood to Recommend Children Eat the Product				4.58	<0.001	0.005
Control	4.67_a_	1.84	733			
Cultured	4.62_ab_	1.86	729			
Cultivated	4.61_ab_	1.87	749			
Cell-Cultivated	4.43_abc_	1.90	729			
Cell-Cultured	4.36_bc_	1.99	734			
Cell-Based	4.32_c_	1.91	711			

Scale: 1 extremely unlikely, 2 moderately unlikely, 3 slightly unlikely, 4 neither likely nor unlikely, 5 slightly likely, 6 moderately likely, 7 extremely likely. Means with the same superscript letter are not significantly different from each other at *p* < 0.05 using the Tukey HSD post hoc Test.

### Criterion E—Be seen as an appropriate term

After reading the explanation of the meaning of the term they had been randomly assigned to see on the product packages, the participants were asked how familiar they were with the *idea* of producing just the parts of beef/chicken/salmon that people eat, instead of raising (or catching) them whole and harvesting them. Of the 3,652 participants *not* in a Control condition, 54.4% reported that they were “not familiar at all,” 12.9% “slightly familiar,” 13.9% “moderately familiar,” 9.6% “very familiar,” and 9.1% “extremely familiar” with the idea of producing beef/chicken/salmon products in this way.

They were asked how appropriate the term was for describing this new way of producing just the parts of beef/chicken/salmon that people eat. All the names were judged to be “neither appropriate nor inappropriate” to “slightly appropriate.” There was a main effect of name in judgements of appropriateness of the term (*F*(4,3647)) = 3.802 *p* = 0.004, η_p_^2^ = 0.004). As shown in Table 13, the term “Cultivated” was seen as the least appropriate term and as significantly less appropriate than both “Cell-Cultivated” and “Cell-Based”.

**Table 13:** Appropriateness of term in communicating the idea of producing just the parts of animals that people eat by common or usual name tested.

	*M*	SD	*N*	*F*	*P* value	η^2^
Appropriateness of Term				3.80	=0.004	0.004
Cell-Cultivated	5.14_a_	1.66	729			
Cell-Based	5.13_a_	1.78	711			
Cell-Cultured	5.09_ab_	1.73	734			
Cultured	4.91_ab_	1.79	729			
Cultivated	4.87_bc_	1.82	729			

Scale: 1 extremely inappropriate, 2 moderately inappropriate, 3 slightly inappropriate, 4 neither appropriate nor inappropriate, 5 slightly appropriate, 6 moderately appropriate, 7 extremely appropriate. Means with the same superscript letter are not significantly different from each other at *p* < 0.05 using the Tukey HSD post hoc Test.

The participants who saw a beef product were asked how clear the term was in communicating that the beef was not “Grass-Fed,” and that it was not “Grain-Fed.” Those who saw a chicken product were asked how clear the term was in communicating that the chicken was not “Free-Range,” and that it was not “Raised Indoors.” Those who saw a salmon product were asked how clear the term was in communicating that the salmon was not “Wild-Caught,” and that it was not Farm-Raised.

There was a main effect of name tested on how clear the term was in communicating that the beef products were not “Grass-Fed” (*F*(4,1208)) = 3.875 *p* = 0.004, η_p_^2^ = 0.013) and a main effect of name on clarity that the products were not “Grain-Fed” (*F*(4,1208)) = 3.788 *p* = 0.005, η_p_^2^ = 0.012). Products with the term “Cell-Cultured” were seen as clearer in communicating that the products were not “Grain-Fed” than products labeled as either “Cultivated” or “Cultured” (Table 14).

**Table 14:** Clarity that the beef product is not grain-fed and not grass-fed by common or usual name tested.

	*M*	SD	*N*	*F*	*P* value	η^2^
Grain-Fed				3.88	=0.004	0.013
Cell-Cultured	4.89_a_	1.94	245			
Cell-Cultivated	4.83_ab_	1.87	237			
Cell-Based	4.62_ab_	2.11	239			
Cultivated	4.36_b_	2.10	245			
Cultured	4.33_b_	2.18	242			
Grass-Fed				3.79	=0.005	0.012
Cell-Cultivated	4.86_a_	1.89	237			
Cell-Cultured	4.82_a_	1.94	245			
Cell-Based	4.55_ab_	2.09	239			
Cultivated	4.36_ab_	2.08	245			
Cultured	4.31_b_	2.16	242			

Scale: 1 extremely unclear, 2 moderately unclear, 3 slightly unclear, 4 neither clear nor unclear, 5 slightly clear, 6 moderately clear, 7 extremely clear. Means with the same superscript letter are not significantly different from each other at *p* < 0.05 using the Tukey HSD post hoc Test.

There was no main effect of name tested on how clear the term was in communicating that the chicken products were not “Free-Range” (*F*(4,1222)) = 1.274 *p* = 0.278). All names were judged to be “neither clear nor unclear” to “slightly clear” (*M* = 4.53, SD = 2.06) [Scale: 1 Extremely Unclear to 7 Extremely Clear]. There was similarly no main effect of the name in terms of clarity in communicating that the products were not “Raised Indoors” (*F*(4,1222)) = 1.274 *p* = 0.224) (*M* = 4.43, SD = 2.08).

Each name was seen as equally clear in communicating that the salmon products were not “Wild-Caught” (*M* = 4.60, SD = 2.04) (*F*(4,1212)) = 2.398 *p* = 0.048). There was a main effect of name on clarity in communicating that the products were not “Farm-Raised” (*F*(4,1212)) = 6.128 *p* < 0.001, η_p_^2^ = 0.020). The term “Cultured” was seen as least clear in conveying that the products were not from aquaculture, while the terms containing the word “cell” were seen as clearer in communicating that the products were not “Farm-Raised” (Table 15).

**Table 15:** Clarity that the salmon product is not farm-raised by common or usual name tested.

	*M*	SD	*N*	*F*	*P* value	η^2^
Clarity of term				6.13	<0.001	0.020
Cell-Cultivated	5.01_a_	1.88	252			
Cell-Cultured	4.72_ab_	1.92	247			
Cell-Based	4.69_abc_	2.08	229			
Cultivated	4.34_bc_	2.09	250			
Cultured	4.21_c_	2.13	239			

Scale: 1 extremely unclear, 2 moderately unclear, 3 slightly unclear, 4 neither clear nor unclear, 5 slightly clear, 6 moderately clear, 7 extremely clear. Means with the same superscript letter are not significantly different from each other at *p* < 0.05 using the Tukey HSD post hoc Test.

The participants were asked how clear the name was in communicating that the product they had seen was not made from plants. There was a main effect of name tested (*F*(4,3647)) = 4.322 *p* = 0.002, η_p_^2^ = 0.005). As shown in Table 16, all names tested were seen as “neither clear nor unclear” to “slightly clear”. The term “Cell-Cultured” was seen as clearer in communicating that the products were not plant-based than the terms “Cell-Based,” “Cultivated” and “Cultured.”

**Table 16:** Clarity that the Beef/Chicken/Salmon Product is not made from plants by common or usual name.

	*M*	SD	*N*	*F*	*P* value	η^2^
Clarity of term				4.32	=0.002	0.005
Cell-Cultured	4.78_a_	1.96	734			
Cell-Cultivated	4.75_ab_	1.93	729			
Cell-Based	4.50_b_	2.07	711			
Cultivated	4.49_b_	2.05	749			
Cultured	4.46_b_	2.12	729			

Scale: 1 extremely unclear, 2 moderately unclear, 3 slightly unclear, 4 neither clear nor unclear, 5 slightly clear, 6 moderately clear, 7 extremely clear. Means with the same superscript letter are not significantly different from each other at *p* < 0.05 using the Tukey HSD post hoc Test.

Participants viewing the novel beef/chicken/salmon products were asked how much they agreed or disagreed that they should be sold in the same section of the supermarket as those that are “Grass-Fed and Grain-Fed,” “Free-Range and Raised Indoors,” or “Wild-Caught and Farm-Raised” [Scale: 1 Strongly disagree to 7 Strongly Agree]. Univariate ANOVAs showed no main effects of name tested for beef (*F*(4,1203)) = 1.626 *p* = 0.760), chicken (*F*(4,1212)) = 1.87 *p* = 0.114), or salmon products (*F*(4,1212)) = 1.506 *p* = 0.198). The participants “Neither Agree nor Disagree” to “Somewhat Agree” that the beef (*M* = 4.51, SD = 1.86), chicken (*M* = 4.32, SD = 1.89), and salmon products (*M* = 4.49, SD = 1.85) should be sold in the same section of the supermarket as their conventional counterparts.

### Consumer perceptions after learning the meaning of the term

After learning the meaning of the term they had seen, the participants were shown the same package a final time, and asked for their overall reactions, their interest in tasting the product, and the likelihood they would purchase the product in the next 6 months if it were available in their grocery store. A MANOVA examining these dependent measures found that there were no main effects of name tested after explaining its meaning, *F*(32, 13329) = 1.136, *p* = 0.274; Wilk’s Λ = 0.990, η_p_^2^ = 0.003. This suggests that any potential marketing advantages a name may initially have are likely to disappear after consumers achieve greater awareness and understanding of the products and the technology used to produce them.

**Fig. 1:**
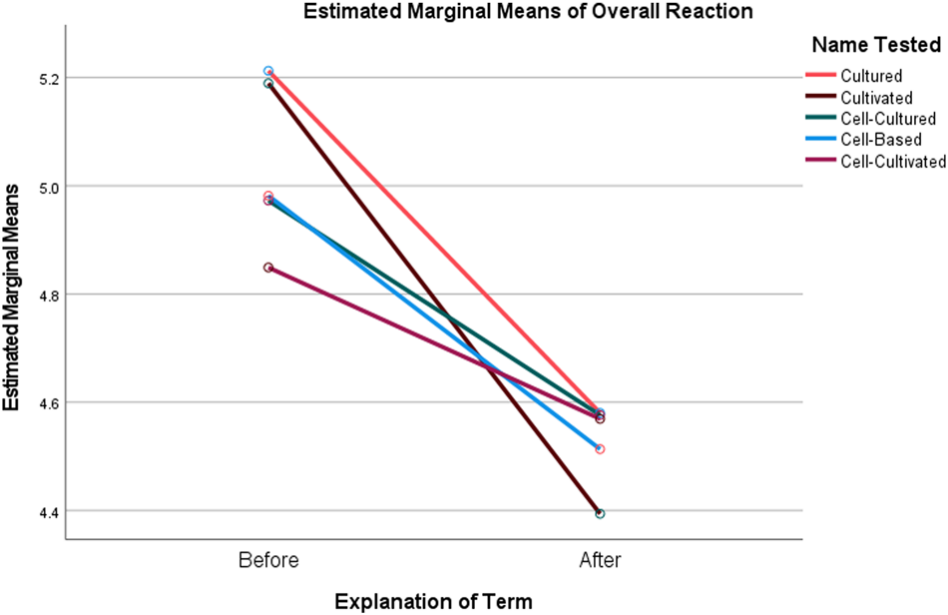
Plot of Marginal Means of Overall Reaction to Products Before and After Explanation of the Terms by Common or Usual Name.

**Fig. 2:**
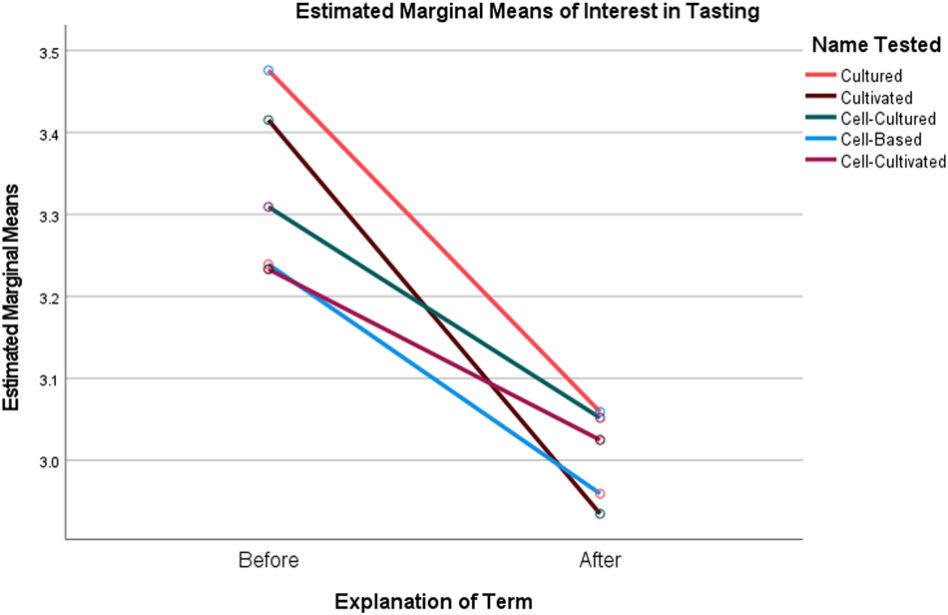
Plot of Marginal Means of Interest in Tasting the Products Before and After Explanation of the Terms by Common or Usual Name.

**Fig. 3:**
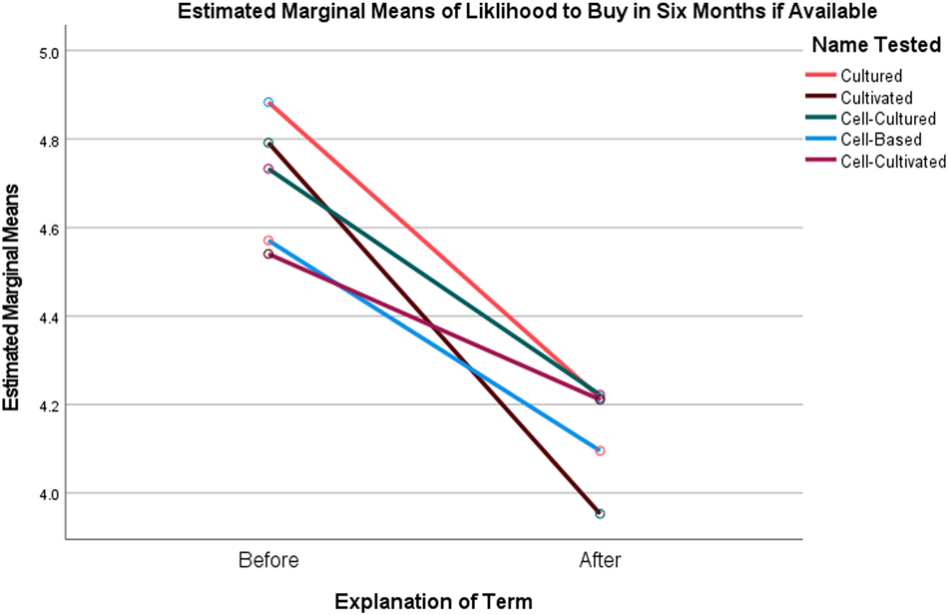
Plot of Marginal Means of Likelihood to Purchase the Products Before and After Explanation of the Terms by Common or Usual Name.

To illustrate this, a repeated measures ANOVA showed a main effect of explaining the term on overall reactions (*F*(1,3647)) = 400.752 *p* < 0.001, η_p_^2^ = 0.099), with significant declines in how positive the participant’s reactions were to the products after reading the explanation (Fig. 1). There was also an interaction effect of the explanation and the name the participant saw (*F*(4,3647)) = 15.15 *p* < 0.001, η_p_^2^ = 0.014). Prior to the explanation, the overall ratings for products with the term “Cultivated” were significantly higher than those for products with the other names (Table 6). After the explanation, overall ratings for products labeled with the term “Cultivated” dropped to the lowest measured among the names (though not significantly different from them). The same pattern was observed with respect to the main effect of the explanation on interest in tasting the products (*F*(1,3647)) = 308.323 *p* < 0.001, η_p_^2^ = 0.078), and the interaction effect of the explanation and the name the participant viewed (*F*(4,3647)) = 7.065 *p* < 0.001, η_p_^2^ = 0.008) (Fig. 2). This pattern was repeated in the main effect of the explanation on likelihood of purchasing the product in 6 months if available in the participant’s grocery store (*F*(1,3647)) = 416.206 *p* < 0.001, η_p_^2^ = 0.102), and the interaction effect of the explanation and the name the participant viewed (*F*(4,3647)) = 10.032 *p* < 0.001, η_p_^2^ = 0.011) (Fig. 3). Interest in tasting and likelihood to purchase products labeled as “Cultivated” dropped significantly more after reading the explanation than was the case for the other terms.

## Discussion

As expected, consumer awareness of the *concept* of producing just the parts of beef/chicken/salmon that people eat, instead of raising (or catching) them whole and harvesting them, remains very low. Most participants (67.4%) reported that they were “not familiar at all” or only “slightly familiar” with the idea. Therefore, the common or usual name chosen must convey significant new information to uninformed consumers. This includes communicating “the basic nature of the food or its characterizing properties or ingredients” (21CFR102.5), and what distinguishes it from other foods. The name chosen must also do so on its own; without the benefit of additional explanatory labeling text or other supporting materials.

None of the names tested in this experiment are part of existing labeling schemes for beef, chicken, or salmon products. Although the methods of production (“Wild-Caught” and “Farm-Raised”) are required to appear on seafood labels (7 CFR § 60.300), no similar regulations for labeling the methods of livestock production exist for meat and poultry products. Labels such as “Grass-Fed” and “Free-Range” are voluntary and the products bearing them are often sold at a price premium, with each making up about one percent of the market for beef or chicken. Therefore, the signal sent by the proposed names that this new method of production is different from conventional beef, chicken, and salmon is likely contingent on a consumer’s familiarity with the existing labels for those conventional products.

It is instructive to examine the Control conditions for each product. In the U.S., conventional salmon products are required to be labeled as either “Wild-Caught” or “Farm-Raised.” Yet, the inclusion of no common or usual name (the Control condition) leads many consumers to assume that the products are “Wild-Caught.” This may be their default assumption if products are not specifically labeled as “Farm-Raised.” The data from this experiment shows that introduction of the term “Cultivated” and the absence of the “Wild-Caught” label signals that the products are “Farm-Raised.”

Responses to the Control condition for Beef Filets and Beef Burgers, which represent the way that most “Grain-Fed” beef is packaged in the U.S., contrast the results for salmon. The default assumption appears to be that in the absence of a label that specifically indicates that the products are “Grass-Fed” or “Grain-Fed,” the products are *neither*, suggesting that most Americans are unaware that when a beef product is not labeled as “Grass-Fed,” it is almost certainly “Grain-Fed.” When “Cultivated” is on the label of the Beef Filets, it is mistaken by one-third of consumers as meaning that the product is “Grass-Fed.” Yet, when selecting Beef Burgers, consumer choice between “Grass-Fed” and “Grain-Fed” beef may not be as salient, so any additional label that consumers have not seen before may signal that the product is different from those they usually purchase. This would likely explain why all the names tested performed similarly in signifying that the Beef Burgers were “Neither Grass-Fed nor Grain-Fed.”

In the Control condition for Chicken Breasts and Chicken Burgers, the absence of a label that specifically indicates that the products are “Free-Range” or are “Raised Indoors,” appeared to cue a default assumption that the product was neither (though it should have signaled that the chickens were “Raised Indoors”). Yet, “Cultured” and “Cultivated” performed more poorly than the control in signaling that the Chicken Burgers were “Neither Free-Range nor Raised Indoors.” Though not measured in this study, it is likely that widespread lack of familiarity with conventional (indoor) chicken production systems by consumers^37^^,^^38^ and their lack of exposure to labeled “Free-Range” alternatives in supermarkets made those categories less salient to the participants.

Given the significant success of plant-based meats in the U.S.^39^, the other salient category for consumers across beef, chicken, and salmon products may be “Plant-Based.” Across the products analyzed, “Cell-Cultured” and “Cell-Cultivated” were viewed as clearest in conveying that the products were not plant-based.

The results of this study are consistent with Hallman & Hallman^13^ in showing that the common or usual names “Cultured” and “Cultivated” inadequately communicated to consumers that the Salmon Fillets and Salmon Burgers presented were different from “Wild-Caught” and “Farm-Raised” Salmon, signaling instead to many consumers that the products were “Farm-Raised.” They therefore fail to meet Criterion A, suggesting that adoption of the terms “Cultured” and “Cultivated” would be problematic with respect to the implementation of a single name across meat, poultry, and seafood products. The results are also consistent with Hallman & Hallman^13^ in showing that terms containing the word “cell” (“Cell-Based,” “Cell-Cultured,” “Cell-Cultivated”) were more effective in conveying to consumers that the products with those labels are different from conventional seafood products.

The participants were least confident in their answers regarding whether the beef, chicken or salmon product was different from conventional products when they saw them labeled as “Cultured” or “Cultivated.” The terms “Cultivated” and “Cultured” were also seen as least clear in communicating that the beef and salmon products were different from their conventional counterparts. Consistent with this, the term “Cultivated” inadequately conveyed that the Beef Filets were different from conventional products, signaling to more than one-third (33.9%) that the products were “Grass-Fed.” These results suggest that “Cultivated” may be misleading to consumers, failing to meet Criterion A for both beef and salmon products. Though not tested in conjunction with shellfish products in this experiment, the term “Cultured” is also commonly used to describe the production of farmed shellfish, making it an inappropriate term to identify shellfish that have not been farmed. “Cultured” is also used to identify fermented dairy products, which may also be problematic to the adoption of a single term across all protein products. As already noted, neither “Cultivated” nor “Cultured” is currently an acceptable term to describe the production of fish/shellfish on a seafood product label (7 CFR § 60.300).

The results reported indicate that people tend to think that being allergic to salmon is considerably more dangerous than being allergic to beef or chicken, which is not true when anaphylaxis results from exposure to any of the proteins. However, each name appropriately signaled to consumers that if they are allergic to beef/chicken/salmon it is “somewhat” to “moderately unsafe” to eat these novel products, meeting Criterion B. The results also show that there is no interaction effect between name tested and protein tested. This is important because it indicates that each of the names is as effective in signaling allergenicity regardless of the protein. Had there been an interaction effect, this would have been a key issue for regulators.

However, while the effect size was small, in comparison to the Control, the addition of a common or usual name to the label signaled that the product was slightly safer for those allergic to eat. This suggests that allergen warnings may need to be highlighted on packages of products made directly from the cells of animals. It should also be noted that each of the products seen by participants included the name of the protein (beef/chicken/salmon) to which someone might be allergic. While not tested in this study, failing to include the name of the protein on the label might mask the fact that the product contains allergens specific to that protein.

None of the names were perceived as inappropriate for communicating the idea of producing just the parts of animals that people eat instead of raising (or catching) them whole and harvesting them, meeting Criterion E. While the difference was small, the term “Cultivated” was seen as least appropriate.

By design, none of the names evaluated was disparaging of other products (Criterion C), and the coding of the open-ended responses after seeing the products did not reveal a clear pattern of negative thoughts, images, or feelings associated with any of the names tested. Before learning the meaning of the names, the participants were slightly to moderately interested in tasting all the products, but least interested in tasting the products labeled as “Cell-Based” and “Cell-Cultivated.” The participants reported that they were “neither likely nor unlikely” to “slightly likely” to purchase the products. They reported that they were least likely to purchase those labeled as “Cell-Cultivated,” and least likely to serve “Cell-Based” products to guests.

None of the names tested suggested to the participants that the products were unsafe to eat or unnatural, although those labeled as “Cell-Cultivated,” “Cell-Based,” and “Cell-Cultured” were seen as less natural than those labeled as “Cultivated” or “Cultured.” No organic standards exist for these products, and none of the names implied that the products were likely to be organic.

However, products labeled with “Cell-Cultivated,” “Cell-Based,” and “Cell-Cultured” were seen as more likely to be genetically modified. Some production methods for these products may rely on inputs that are genetically modified^40^. However, those products that do not involve genetic modification may wish to provide additional labeling indicating this, as may be permitted by regulation^41^.

None of the names tested influenced perceptions of how nutritious or how healthy the products were. All were perceived as moderately nutritious and as neither healthy nor unhealthy. Each product was imagined tasting slightly to moderately good, though those labeled as “Cultured” were thought to taste slightly better than those labeled as “Cell-Based.” The participants were more likely to recommend that people who are pregnant eat the Control products than the “Cell-Cultured,” “Cell-Based” and “Cell-Cultivated” products. They were also more likely to recommend that children eat the Control products than those labeled as “Cell-Cultured” and “Cell-Based.” They were also not opposed to selling the novel beef, chicken, and salmon products in the same section of the supermarket as their conventional counterparts. Therefore, each name meets Criterion D.

The results also show that after reading an explanation of the meaning of the terms, the main effects of the common or usual name on key consumer acceptance measures disappear, including overall reactions to the product, interest in tasting, and likelihood to purchase the products if available in their grocery store. This suggests that once consumers have greater familiarity and understanding of these novel products and the processes used to create them, any of the initial marketing advantages of the names “cultivated” and “cultured” (some of which may be based on consumer misperceptions that they are conventional products) are likely to vanish. While it is difficult to make definitive conclusions based on pre-post measures in a 12 min online experiment, the differences between initial overall reactions to products labeled with these terms and those after the explanation of the term may indicate the possibility of a consumer “backlash” related to learning that their initial perceptions of the nature of the product was incorrect. Not directly measured in this experiment, this effect may be worthy of future research.

In choosing the best name among “Cell-Based,” “Cell-Cultured” and “Cell-Cultivated,” it should be noted that the differences in the means of many of the key dependent measures and their associated effect sizes are quite small. There were no statistically significant differences among the three in their ability to signal that the products were different from conventional products or in signaling allergenicity.

There were also no statistically significant differences among the three terms with respect to key consumer acceptance measures, including: initial overall reactions, interest in tasting, likelihood to purchase in a grocery store, likelihood to order in a restaurant, likelihood to serve to guests, likelihood to recommend that pregnant women and children eat the products, perceived appropriateness of the term, perceived clarity in communicating that the product is different from conventional products, and is not made from plants. The best name of the three is likely to be the one that achieves consensus across the meat, poultry, and seafood sectors and is adopted and actively promoted by all, thereby coming into common use by the industry, regulators, consumer and environmental organizations, and by the media.

However, examining its ranked position with respect to both the Control conditions and those of “Cell-Based” and “Cell-Cultivated,” the overall pattern of results across the beef, chicken, and salmon products suggests that the term “Cell-Cultured” may be most advantageous to adopt. It performs well in communicating that the products are different from conventional products and in communicating allergenicity, thereby meeting the two key regulatory criteria. With respect to consumer acceptance, the participants indicated that they are as interested in tasting “Cell-Cultured” products, as likely to purchase them in a grocery store, as likely to order them in a restaurant, and as likely to serve them to guests as the Control products. Given that meat and poultry products in the US are not required to have labels declaring their production methods, the Control packages shown in this study represent packages of conventional meat without any voluntary labeling with respect to production method. It is against those conventional meat and poultry products which the “Cell-Cultured” products would compete.

As with any experiment, this study has limitations. The participants saw high-resolution images of the products and were not able to physically interact with them as they would in a grocery store. The packages were also seen in isolation, without the context of having other products on the same grocery shelf to which they might be compared. Tests of the packages and common or usual names under realistic shopping conditions would add to the strength of this study.

In summary, the results across the six beef, chicken, and salmon products suggest that the common or usual names “Cultivated” and “Cultured” do not signal to many consumers that the novel products are different from conventional products, failing to meet a key regulatory criterion that a common name not be misleading. The three names containing the word “cell,” “Cell-Based,” “Cell-Cultured,” and “Cell-Cultivated” met the two regulatory criteria against which they were measured and were not significantly different on most consumer perception measures. However, the overall pattern of results suggests that the term “Cell-Cultured” may have a slight edge with respect to consumer acceptance. Overall, the participants were as interested in tasting them, in purchasing them, and in ordering them in a restaurant, and as likely to serve them to guests as they were the Control products without any common or usual name. This suggests that the term “Cell-Cultured” may be the best common or usual name for meat, poultry, and seafood products made directly from the cells of animals.

## Methods

### Experimental design

A 6 × 6 full factorial design was used to test the main and interaction effects between 6 names (Cell-Based, Cell-Cultured, Cell-Cultivated, Cultured, Cultivated, and control—just the product with no common or usual name as a modifier). These were associated with 3 products (beef, chicken, salmon) presented in 2 forms (whole cuts or burgers). Each participant was randomly assigned to one of the 36 conditions and saw only one term tested on a single product shown in one form.

### Materials

**Fig. 4:**
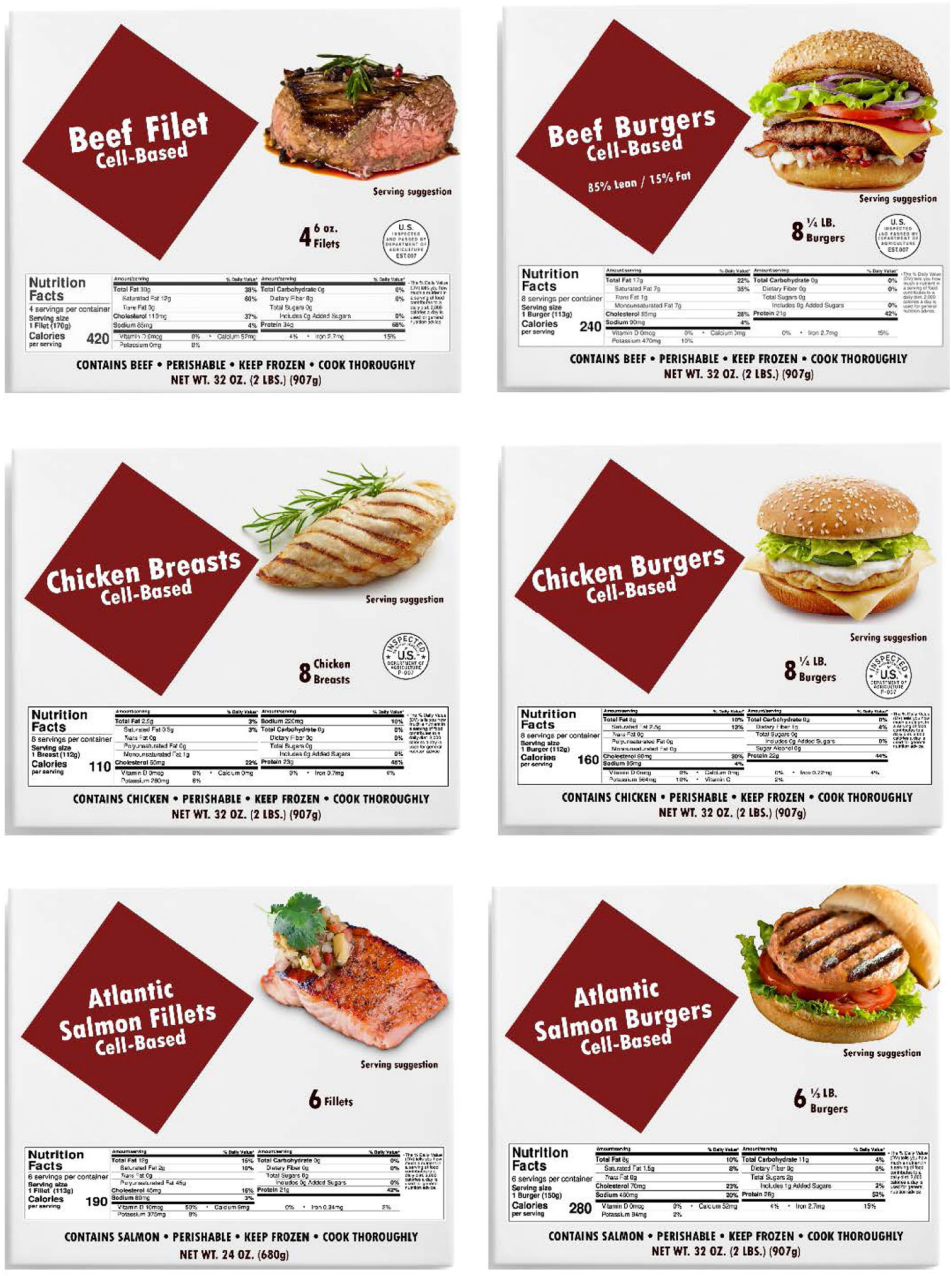
Package Images Using Cell-Based Nomenclature as an Example.

High-resolution pictures of the front of boxes containing frozen Beef Filets, Beef Burgers, Chicken Breasts, Chicken Burgers, Atlantic Salmon Fillets, and Salmon Burgers were created for this experiment, based on packages of similar conventional products currently available in U.S. supermarkets. The name of the product and the common or usual name to be evaluated was placed in a maroon diamond on the left side of the package. The common or usual name tested was printed directly below the product name (Fig. 4) in text half the size of the product name. On the right side of each package, a picture of the cooked product was presented as a “serving suggestion.” Below it, the number of beef, chicken, or salmon burgers, chicken breasts, beef filets, or salmon fillets in the package was listed, along with their weights. The packages containing beef and chicken products also displayed an appropriate (but fictitious) USDA inspection mark. Although a Nutrition Facts Label (NFL) normally appears on the back or side of food packages, to make the information easily accessible to the participants, an NFL was shown in the bottom third of the package, showing accurate values obtained from conventional products. The net weight was printed at the bottom of the package along with declarations that the product “CONTAINS BEEF,” “CONTAINS CHICKEN,” or “CONTAINS SALMON,” the product is “PERISHABLE,” and advising consumers to “KEEP FROZEN” and to “COOK THOROUGHLY.”

### Participants

Data was collected between November 15 and November 28, 2021. The study participants consisted of 4385 adult American consumers (18 and older) recruited from the Dynata.com web-based consumer panel using quota sampling to match a frame derived from the 2018 American Community Survey (ACS).

### Procedure

The procedures used were adapted from those reported in Hallman and Hallman^13,14^ and were approved by the Institutional Review Board of Rutgers University (Pro2021002262 11/15/21).

The participants indicated their age, and after reading an informed consent statement, consented to participate by explicitly choosing the “yes” option when asked “Do you agree to participate in this study?” Each participant was then randomly assigned to one of the 36 beef, chicken, or salmon conditions. They were asked how often they had eaten a meal containing that protein in the previous 12 months. If they had not eaten any, they were asked to indicate why.

To ensure that the participants carefully considered the packages, each was shown three times. To measure perceptions relevant to Criteria C and D, the participants were asked to carefully examine the package a first time, to write the “first thought, image, or feeling that comes to mind when seeing this package” using an open-ended response option, and then to rate how positive or negative their response was. The participants were shown the same package again and were asked to record their second thought, image, or feeling, and to rate how positive or negative it was. After being shown the package a third time, they rated their overall reactions to the product, and indicated how interested they would be in tasting it, how likely they would be to purchase it in the next 6 months if it were sold in their grocery store, how likely they would be to order it in a restaurant in the next 6 months, and how likely they would be to serve the product to guests. They were also asked how likely they would be to search for more information about the product on the Internet, their familiarity with Quick Response (QR) codes, and how likely they would be to scan a QR code to find more information about the products if one were printed on the product package.

After being shown an enlarged image of the “serving suggestion” that appeared on the package, the participants were asked how familiar they were with the product in general (e.g., Beef Filets), if they had ever tasted it, and if so, how much they liked the taste. They indicated whether they had ever ordered the products in a restaurant, and how likely they would be to do so in the next 6 months. Similarly, they reported whether they had ever purchased the uncooked product in a store or online and how likely they would be to do so in the next 6 months. They were asked whether they had ever cooked the product, and whether they or anyone else in their household is allergic to the product.

To assess Criterion A, the participants viewed an enlarged image of the diamond with the product name and the common or usual name to be tested. They were then asked, “Which of the following best describes this beef/chicken/salmon?” The response categories for the beef products were “Grass-Fed,” “Grain Fed,” and “Neither Grass-Fed nor Grain-Fed.” For the chicken products, the categories were, “Free-Range,” “Raised Indoors,” and “Neither Free-Range nor Raised Indoors.” For Salmon, the response categories were “Wild-Caught,” “Farm-Raised,” and “Neither Wild-Caught nor Farm-Raised.” They were also asked how confident they were in their response.

To evaluate Criterion B, participants were asked how safe it would be for those allergic to beef/chicken/salmon to eat the product, as well as how safe it would be to consume the product if one is not allergic to beef/chicken/salmon. They then rated how natural they perceived the product to be, how likely it is that the product is organic, and how likely that it had been genetically modified.

An enlarged image of the product’s Nutrition Facts Label was displayed and as measures of Criterion D, the participants asked to rate how nutritious it is, how healthy it is, and how good or bad it likely tastes. Then they were asked how likely they would be to recommend that pregnant women eat the product, and how likely they would be to recommend that children eat it. In addition to being seen as particularly vulnerable populations, both pregnant women and young children are advised to limit their intake of fish that are high in methylmercury^42^. While salmon typically contains very low levels of methylmercury (and is considered a “best choice” for consumption), seafood produced directly from the cells of fish would likely be “free-from” methylmercury and other contaminants associated with “Wild-Caught” and “Farm-Raised” fish. This may make them a valued alternative to those seeking the nutritional benefits of fish, but without the potential risks associated with them.

A common or usual name must convey appropriate meaning on its own, without additional explanatory text on the label. Therefore, no explanation of the common or usual name shown to the participant was provided prior to the last segment of the experiment. Participants not in the control conditions then read the following description of the term they saw, with the appropriate common or usual name, protein, and product name inserted in each explanatory paragraph:

“The term {common or usual name} indicates that this {Beef/Chicken/Salmon} differs from both {Grass-Fed and Grain-Fed Beef / Free-Range Chickens and Chickens Raised indoors on a farm/Wild-Caught and Farm-Raised Salmon}. It tastes, looks, and cooks the same and has the same nutritious qualities as {Beef/Chicken/Salmon} produced in traditional ways. Yet, it involves a new way of producing just the parts of {Beef/Chicken/Salmon} that people eat, instead of (catching or) raising them whole and harvesting them. {common or usual name} means that a small number of cells from selected {cattle/chickens/Atlantic salmon were placed in a nutrient solution, where they grew and reproduced many times. The resulting meat was then formed into {Filets/Breasts/Fillets/Burgers} that can be cooked and enjoyed in the same way as other {Beef/Chicken/Salmon} products.”

The participants were asked to indicate their familiarity (before the survey) with “the *idea* of producing just the parts of {Beef/Chicken/Salmon} that people eat, instead of (catching or) raising them whole and harvesting them.” As a measure of Criterion E, they were asked to indicate how appropriate the term was for describing this idea. Those who saw beef products then rated the clarity of the term in communicating that the product “was not Grass-Fed” and “was not Grain-Fed.” Those who viewed chicken products rated the term’s clarity in conveying that the product “was not Free-Range” and “was not Raised Indoors,” while those who saw salmon products rated the clarity of the term in signaling that the product “was not Wild-Caught” and “was not Farm-Raised.” All participants were then asked how clear the term was in communicating that the product was not made from plants and how much they agreed or disagreed that the products they viewed should be sold in the same section of the supermarket as {Grass-Fed and Grain-Fed Beef; Free-Range and Chickens Raised Indoors; Wild-Caught and Farm-Raised Salmon}.

After having read the description of the common or usual name to which they had been randomly assigned, the participants were shown the associated package a final time. Repeating questions posed prior to learning about the meaning of the term, they were asked how positive or negative their overall reaction to the product was and how interested they would be in tasting it. They were then asked about their likelihood of (1) purchasing the product in the next 6 months if sold in their grocery store; (2) ordering it if seen on a restaurant menu; and (3) serving it to guests in the next 6 months. Finally, they asked to rate how natural the product is, how likely it is to be organic, and how likely it is genetically modified. These constructs are related to each other and often serve as heuristics predictive of consumer food choices and preferences^43–45^.

The participants then answered questions related to an unrelated second experiment, which is not reported here. The participants finished by reporting how well they could see the images presented in the study and then answered a set of standard demographic questions.

### Statistical analyses

Analyses were conducted on unweighted data using IBM SPSS Statistics for Windows (version 27; IBM Corp., Armonk, New York). Differences in means were tested using Analyses of Variance (ANOVA) to generate effect sizes using partial eta-squared (η_p_^2^). A *p* value of 0.01 was used to identify statistically significant main effects and interactions. Differences in proportions were analyzed using *Z*-tests of column proportions with Bonferroni correction. A *p* value of 0.05 was used to determine statistically significant differences.

### Reporting summary

Further information on research design is available in the Nature Research Reporting Summary linked to this article.

### Supplementary information

Supplemental Information

Reporting Summary

## Data Availability

The data analyzed as part of the current study are available from the corresponding author on reasonable request.
