# Supplementary material for: Cell-based, cell-cultured, cell-cultivated, cultured, or cultivated. What is the best name for meat, poultry, and seafood made directly from the cells of animals?
Source: NPJ Sci Food. 2023 Dec 6;7:62. doi: 10.1038/s41538-023-00234-x (PMC10700563; doi:10.1038/s41538-023-00234-x)
Supplement: Supplementary file 1 — Supplemental Information [file 41538_2023_234_MOESM1_ESM.docx]

**Supplementary Information**

**Supplementary Table 1**

*Open-Ended First Thoughts, Images, and Feelings Categorized*

|  | Name Tested | | | | | | Total |
| --- | --- | --- | --- | --- | --- | --- | --- |
|  | Cell-Based | Cell-Cultured | Cell-Cultivated | Cultured | Cultivated | Control |  |
| None/IDK | 5.9% | 6.5% | 5.3% | 7.1% | 5.5% | 6.4% | 6.1% |
| Delicious/Appetizing/Yum/Want to Eat/Try/Buy | 15.3% | 15.9% | 15.9% | 19.5% | 19.9% | 20.6% | 17.9% |
| Amazing/Awesome/ Attractive/Cool/Good/Great/ Like it/Love it | 16.9% | 19.2% | 16.7% | 19.8% | 19.5% | 20.2% | 18.7% |
| Ok/ Acceptable/Normal | 2.0% | 2.9% | 2.2% | 1.9% | 1.9% | 2.0% | 2.1% |
| Bad/Disgusting/ Yuk Unappetizing/Unappealing | 7.6% | 7.2% | 6.9% | 6.3% | 7.5% | 8.2% | 7.3% |
| Artificial/Fake/Not Natural/ Lab Grown/Manufactured | 2.3% | 5.0% | 3.8% | 1.1% | 2.4% | 0.8% | 2.6% |
| Processed | 0.7% | 0.4% | 0.8% | 1.1% | 0.5% | 0.4% | 0.7% |
| Real or Fake? | 1.1% | 0.7% | 0.3% | 0.5% |  | 0.3% | 0.5% |
| Sustainable/Environmentally friendly | 0.3% |  |  |  |  | 0.1% | 0.1% |
| GMO/"Frankenfood"/Cloned | 0.7% | 0.4% | 0.4% |  |  |  | 0.3% |
| Plant-Based | 1.1% | 0.5% | 0.4% |  | 0.3% |  | 0.4% |
| Concerned/Worried/ Unhealthy/Bad for you | 1.7% | 1.5% | 1.1% | 0.8% | 1.2% | 1.4% | 1.3% |
| Common Name Question (e.g., What does Cell-Cultured Mean?) | 5.5% | 2.7% | 6.2% | 1.5% | 1.2% |  | 2.8% |
| Common Name | 4.1% | 2.0% | 4.4% | 0.8% | 1.3% |  | 2.1% |
| Meat/Seafood name ("Salmon") | 6.9% | 6.5% | 5.2% | 6.0% | 5.5% | 6.5% | 6.1% |
| Meat/Seafood preparation ("grilled") | 3.2% | 4.9% | 4.9% | 5.3% | 4.5% | 5.0% | 4.7% |
| Nutritional Aspects ("High protein") | 3.0% | 2.7% | 2.5% | 3.3% | 4.1% | 3.7% | 3.2% |
| Healthy/Good for You/Natural/Organic | 3.8% | 4.0% | 3.0% | 4.3% | 3.9% | 4.0% | 3.8% |
| Question/Confusion | 2.3% | 2.7% | 2.3% | 3.0% | 1.7% | 1.9% | 2.3% |
| Curious/Interesting | 2.3% | 1.6% | 2.7% | 1.1% | 1.2% | 1.6% | 1.8% |
| New/Innovative/ Unfamiliar/Different | 2.0% | 2.0% | 1.9% | 1.1% | 1.5% | 0.8% | 1.6% |
| Do Not Like/Eat meat/fish/seafood | 0.8% | 0.4% | 1.0% | 0.7% | 1.1% | 1.9% | 1.0% |
| Frozen/Not Fresh | 1.1% | 1.0% | 1.6% | 2.2% | 2.1% | 1.4% | 1.6% |
| Not Wild |  |  | 0.1% | 0.3% | 0.8% | 0.4% | 0.3% |
| Fresh | 0.6% | 0.7% | 1.8% | 0.4% | 0.5% | 1.0% | 0.8% |
| Basic/Generic/Blah/Bland/ Boring/Packaging Negative/General | 2.5% | 1.4% | 1.8% | 2.2% | 3.3% | 2.2% | 2.2% |
| Packaging "Positive"/Clean/ Simple/Convenient | 1.3% | 1.8% | 1.4% | 1.9% | 2.0% | 1.0% | 1.6% |
| Portion Size/Quantity | 1.0% | 0.8% | 0.4% | 1.8% | 1.5% | 1.1% | 1.1% |
| Expensive/High Quality | 1.1% | 0.7% | 0.8% | 1.5% | 1.1% | 2.3% | 1.3% |
| Cheap/Inexpensive | 0.4% | 0.4% | 0.3% | 0.4% | 0.4% | 0.3% | 0.4% |
| Food/Meal/Company/Brand | 1.4% | 1.8% | 2.9% | 2.6% | 1.6% | 2.7% | 2.2% |
| Other | 1.3% | 1.5% | 0.8% | 1.4% | 2.0% | 1.8% | 1.5% |
|  | 100.0% | 100.0% | 100.0% | 100.0% | 100.0% | 100.0% | 100.0% |

**Supplementary Table 2**

*Open-Ended Second Thoughts, Images, and Feelings Categorized*

|  | Name Tested | | | | | | Total |
| --- | --- | --- | --- | --- | --- | --- | --- |
|  | Cell-Based | Cell-Cultured | Cell-Cultivated | Cultured | Cultivated | Control |  |
| None/IDK | 6.6% | 6.4% | 6.7% | 8.1% | 6.9% | 8.0% | 7.1% |
| Delicious/Appetizing/Yum/Want to Eat/Try/Buy | 13.6% | 13.9% | 10.8% | 12.8% | 13.2% | 13.5% | 13.0% |
| Amazing/Awesome/ Attractive/Cool/Good/Great/ Like it/Love it | 10.1% | 11.0% | 10.0% | 12.9% | 11.9% | 10.1% | 11.0% |
| Ok/ Acceptable/Normal | 1.4% | 1.2% | 1.5% | 0.8% | 1.1% | 0.5% | 1.1% |
| Bad/Disgusting/ Yuk Unappetizing/Unappealing | 5.5% | 6.0% | 6.6% | 6.2% | 6.0% | 6.0% | 6.0% |
| Artificial/Fake/Not Natural/ Lab Grown/Manufactured | 2.3% | 4.4% | 4.1% | 0.8% | 1.6% | 1.1% | 2.4% |
| Processed |  | 0.5% | 0.1% | 0.1% | 0.4% | 0.1% | 0.2% |
| Real or Fake? | 1.1% | 0.7% | 1.1% | 0.5% | 0.7% | 0.1% | 0.7% |
| Sustainable/Environmentally friendly | 0.3% | 0.3% | 0.4% | 0.1% | 0.4% | 0.1% | 0.3% |
| GMO/"Frankenfood"/Cloned | 0.6% | 0.5% | 0.5% | 0.1% |  |  | 0.3% |
| Plant-based | 0.6% | 0.1% | 0.4% |  |  |  | 0.2% |
| Concerned/Worried/ Unhealthy/Bad for you | 1.8% | 1.0% | 1.4% | 0.8% | 2.0% | 1.4% | 1.4% |
| Common Name Question | 9.7% | 7.4% | 8.2% | 4.1% | 3.2% | 0.3% | 5.5% |
| Common Name | 5.5% | 5.0% | 7.0% | 3.3% | 3.6% | 0.3% | 4.1% |
| Meat/Seafood name ("Salmon") | 3.8% | 3.1% | 4.0% | 3.6% | 2.9% | 5.0% | 3.7% |
| Meat/Seafood preparation ("grilled") | 3.8% | 3.4% | 4.7% | 6.6% | 5.9% | 5.6% | 5.0% |
| Nutritional Aspects ("High protein") | 9.3% | 9.1% | 8.9% | 10.7% | 9.1% | 10.4% | 9.6% |
| Healthy/Good for You/Natural/Organic | 4.9% | 4.6% | 4.4% | 4.8% | 5.2% | 5.5% | 4.9% |
| Question/Confusion | 3.1% | 4.8% | 2.9% | 3.7% | 4.4% | 4.5% | 3.9% |
| Curious/Interesting | 1.1% | 1.4% | 1.8% | 0.3% | 1.3% | 0.7% | 1.1% |
| New/Innovative/ Unfamiliar/Different | 1.4% | 0.8% | 0.7% | 1.1% | 0.8% | 0.5% | 0.9% |
| Do Not Like/Eat meat/fish/seafood |  | 0.3% | 0.4% | 0.4% | 0.5% | 0.7% | 0.4% |
| Frozen/Not Fresh | 1.5% | 1.1% | 0.5% | 1.5% | 1.3% | 1.9% | 1.3% |
| Not Wild |  |  | 0.3% | 0.4% | 0.5% | 0.1% | 0.2% |
| Fresh | 0.6% | 0.5% | 0.3% | 0.5% | 0.9% | 1.6% | 0.8% |
| Basic/Generic/Blah/Bland/ Boring/Packaging Negative/General | 3.4% | 2.5% | 2.9% | 3.8% | 2.7% | 4.8% | 3.3% |
| Packaging "Positive"/Clean/ Simple/Convenient | 1.4% | 1.8% | 2.2% | 2.3% | 3.1% | 4.6% | 2.6% |
| Portion Size/Quantity | 2.7% | 3.1% | 2.2% | 3.2% | 4.5% | 6.1% | 3.6% |
| Expensive/High Quality | 1.0% | 1.2% | 0.5% | 1.6% | 1.1% | 1.5% | 1.2% |
| Cheap/Inexpensive | 0.6% | 0.3% | 0.4% | 1.4% | 0.5% | 0.8% | 0.7% |
| Food/Meal/Company/Brand | 0.8% | 1.8% | 1.8% | 1.8% | 2.3% | 2.7% | 1.9% |
| Other | 1.5% | 1.8% | 2.2% | 1.5% | 1.9% | 1.2% | 1.7% |
|  | 100.0% | 100.0% | 100.0% | 100.0% | 100.0% | 100.0% | 100.0% |
